# Otolith Trace Elemental Analyses of South American Austral Hake, *Merluccius australis* (Hutton, 1872) Indicates Complex Salinity Structuring on their Spawning/Larval Grounds

**DOI:** 10.1371/journal.pone.0145479

**Published:** 2016-01-04

**Authors:** Paul Brickle, Pia C. Schuchert, Alexander I. Arkhipkin, Malcolm R. Reid, Haseeb S. Randhawa

**Affiliations:** 1 Directorate of Natural Resources, Fisheries Department, Falkland Islands Government, Stanley, Falkland Islands; 2 South Atlantic Environmental Research Institute, Stanley Cottage, Stanley, Falkland Islands; 3 School of Biological Sciences, University of Aberdeen, Zoology Building, Tillydrone Avenue, Aberdeen, AB24 2TZ, United Kingdom; 4 School of Biology, Newcastle University, Newcastle upon Tyne, NE1 7RU, United Kingdom; 5 Department of Chemistry, University of Otago, PO Box 56, Dunedin, 9054, New Zealand; 6 Ecology Degree Programme, Department of Botany, University of Otago, PO Box 56, Dunedin, 9054, New Zealand; Department of Agriculture and Water Resources, AUSTRALIA

## Abstract

Trace element signatures of otolith edges and cores from 335 austral hake (*Merluccius autralis*) were analysed using LA-ICPMS from samples collected in Chilean and Falkland Islands' waters, in order to provide potential insights into stock discrimination and migrations. Fish were caught in two locations in Chile and four locations in the south-west of the Falkland Islands Shelf. Univariate and multivariate analyses of trace element signatures in the edges of otoliths, representing adult fish, were not able to distinguish between samples collected in Chile and the Falkland Islands. Cluster analyses based on Ward’s similarity/distance metric suggested that it was possible to identify two groups from core signatures. Further analyses of this perceived clustering of the core concentrations revealed that this was largely due to the wide spread of Sr/Ca ratios in the otoliths’ cores. Gaussian finite mixtures using MCMC methods confirmed that Sr/Ca ratios form two separate distributions with significantly different mean values while concentrations of other elements showed no evidence of the presence of two or more distributions. The results suggest that there is only one spawning stock of austral hake with spawning situated in and around the Chilean fjords (43°30’S– 47°S) and the variation in Sr/Ca ratios likely suggests complex salinity structuring in this area.

## Introduction

The austral hake *Merluccius autralis* (Hutton, 1872) is a benthopelagic species found between the depths of 60 m to 1,000 m in the Falkland Islands [1]. Two distinct geographical populations are recognized, one from New Zealand (New Zealand population) and the other from southern South America (Patagonian population). The New Zealand population occurs around Chatham Rise, Campbell Plateau and South Island northward to the East Cape. The Patagonian population extends from 40°S (Chiloe Island) in the Pacific, southward around the southern tip of South America, to the continental shelf north to 49°S and the slope north to 38°S in the Atlantic. Ho [2] and Inada [3] suggested that the New Zealand population derived from the South American one during an inter-glacial period in the Pleistocene. However, according to Machado-Schiaffino et al [4], based on mtDNA, this occurred earlier with New Zealand and South American stocks separating 50,000 years ago. The last glaciation experienced one of the coldest pulses between interglacial periods in the South Pacific during this time [5] and it is likely that populations living in these regions lost contact. Once the two populations had adapted to local conditions, the opening of the Pacific (*c*. 20,000 years BP; [4]) did not result in increased gene flow between Patagonian and New Zealand hake.

For the South American region, based on parasitological and morphological studies, George-Nascimiento and Arancibia [6] suggested that *M*. *australis* migrate from their inshore spawning grounds at 49°S along the Chilean coast to their feeding grounds, via Cape Horn, in the southern south Atlantic. Arkhipkin et al [7] examined patterns in fisheries and observer data in the Falkland Islands and showed that *M*. *austalis* occurred on the southern shelf of the Falkland Islands south of 51°S and that during the spawning period from July to September they are largely absent, suggesting that fish from both Chile and the South Atlantic represent one inter breeding population. There is some confusion about the spawning grounds in the literature. It is acknowledged that the species’ main spawning area is located along the shelf break and canyons in the northern area of Chilean Patagonia, close to Guafo and Guamblin Islands (43–45◦S), and spawning occurs in austral winter from July through September [8–11]. However, Aguayo and Zuleta [12] and Aguayo et al [13] also indicate a secondary spawning area to the south at 52° - 54°S. Payá and Ehthardt [14] and Bustos et al [15] only acknowledge the Guamblin and Guafo areas as the spawning grounds. According to Payá (pers. comm.) the spawning area in the southern area may have been there in the past (1980s) but not today. However, it is possible that some small and dispersed spawning areas remain, but nothing as significant as the Guafo-Guamblin area in the north.

The aim of this study was to examine the stock structure of *M*. *australis* based on otolith microchemistry. The elemental signatures from the otolith edge provide information on the environment experienced by the fish prior to capture, whereas the otolith core represents the larval and early pelagic juvenile life stages and may contain information on spatial segregation during early life stages.

## Methods

### Ethics Statement

This project was sanctioned by the Falkland Islands Government (FIG). Samples were collected on licenced trawlers operating in the Falkland Islands Conservation Zones. Specimens collected in Chile were from trawlers operating under agreement with the relevant authorities. Deceased individuals captured and destined for this study were labelled and boxed for deep freezing for subsequent analyses on shore.

### Sample collection

**Fig 1:**
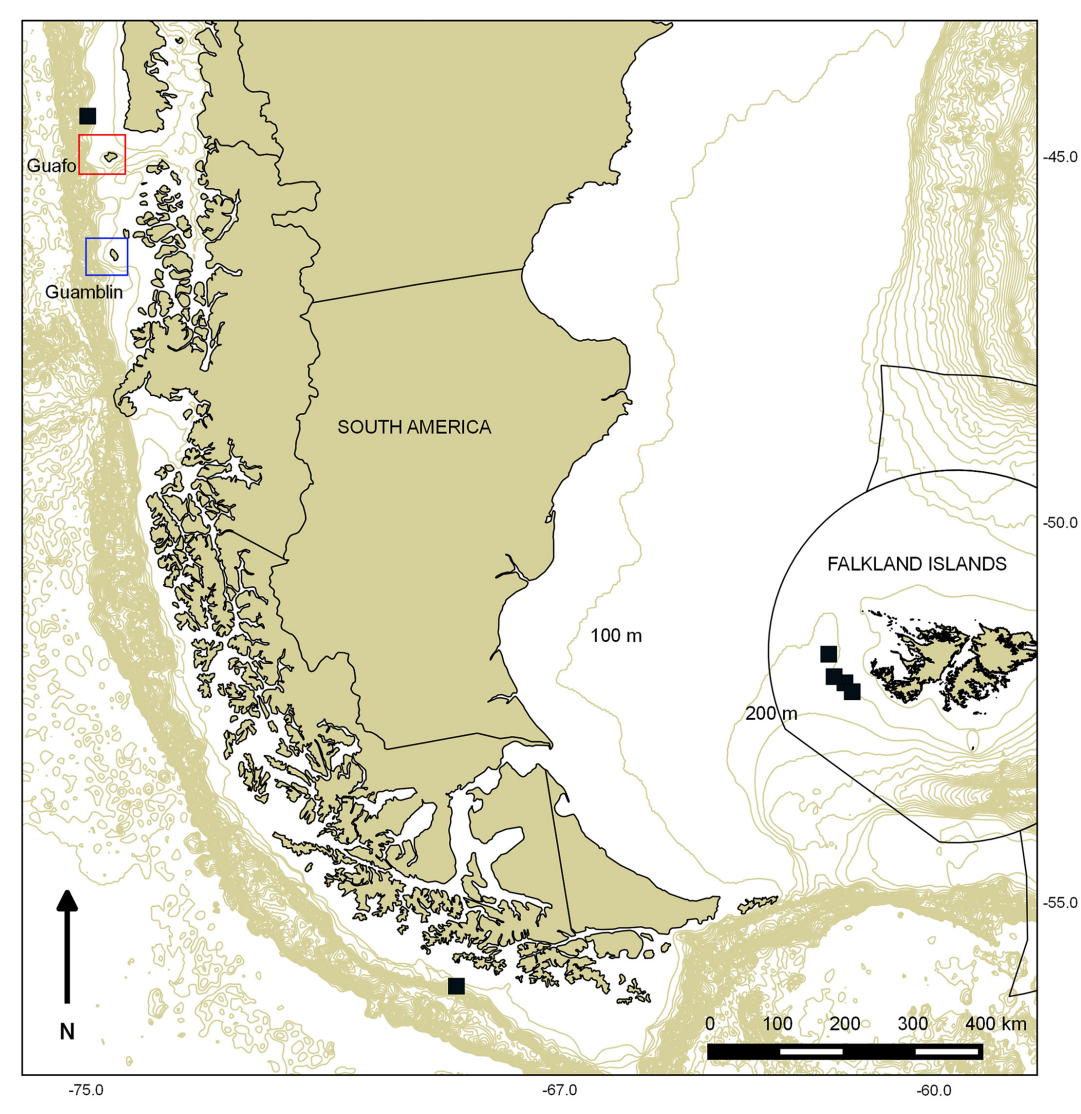
Sampling locations for *Merluccius auatralis* between November 2008 and January 2009. The spawning grounds are located near and around the location of the most northerly sample collection in the Pacific.

Austral hake were collected by trawlers operating in Chile and the Falkland Islands between November 2008 and January 2009. A total of 335 *M*. *australis* (Fig 1, Table 1), 161 from the South Pacific and 174 from the South Atlantic, were collected aboard commercial and research vessels. All of the samples were brought back to the Falkland Islands Government Fisheries Department’s (FIFD) laboratory in Stanley where they were processed. The fish were assessed for sex and maturity, total length (to the nearest cm below; L_T_) and weight (to the nearest g). To reduce possible year-class effects on trace-element composition of otolith cores, the project aimed to collect fish that were restricted to L_T_ of 65–75 cm and their ages were confirmed by FIFD laboratory. Otoliths were collected using plastic forceps and stored dry in plastic Eppendorf vials to avoid any metal contamination. One otolith from each fish was randomly chosen for trace element analysis while the other was retained for ageing. The gills and viscera were also examined for the presence of parasites, and a tissue sample was taken for genetic investigation at a later date.

**Table 1:** Specimen sampled, mean concentration, as ratio to Ca, for Ba, Na, Mg, Sr and Sn in otolith edge, standard deviation and the transformation λ applied (x^λ^), in case of λ = 0, transformation is log10(x).

Location	λ	Atlantic				Pacific			
Sex		M		F		M		F	
N		28		106		37		96	
Length (cm)		47–88		52–88		45–66		43–67	
Mean Length (cm)		70.9		71.0		56.6		57.9	
Age		4–7				3–6			
Mean age (yr)		5.1				4.8			
Concentration		Mean	Std	Mean	Std	Mean	Std	Mean	Std
Ba	0	0.0512	0.0293	0.0419	0.0183	0.0676	0.0316	0.0500	0.0280
Na	-1	0.00788	0.0009	0.00773	0.0010	0.0081	0.0011	0.0079	0.0011
Mg	-1	0.2744	0.1098	0.295	0.1475	0.2782	0.1753	0.2813	0.1194
Sr	0.5	48.509	10.374	43.783	8.410	50.987	7.585	45.879	9.595
Sn	1	0.0155	0.00299	0.1567	0.00270	0.01475	0.0025	0.01544	0.00267

### Trace element analyses

Minor and trace element signatures were examined (by Laser ablation-Inductively coupled plasma mass spectrometry (LA-ICP-MS) at the Centre for Trace Element Analysis housed in the Department of Chemistry, University of Otago (Dunedin, New Zealand). Instrumentation was a New Wave UP213 laser and an Agilent 7500cs quadrupole ICP-MS. Otoliths were mounted on standard glass slides cut to 48 mm x 25 mm and analysed for 30 different elements, concentrations reported as ratios to ^44^Ca. Multiple isotopes of Mg (^24^Mg and ^25^Mg), Ca (^43^Ca, ^44^Ca, ^47^Ca, and ^48^Ca), and Fe (^54^Fe and ^57^Fe) were measured to account for potential interference or spillover, and ^15^N^14^N was monitored to confirm flushout after sample changeover. Argon gas blanks were run for 20 seconds prior to each spot and subsequently subtracted from the corresponding sample values. For each otolith, two spots, corresponding to the core and edge, respectively, were ablated for 40 seconds using a laser spot of diameter 80 µm, with a laser flux of 2.85 to 4.74 mJ cm^-2^ (standard deviation < 5% within a run), leaving a crater typically 74 µm in depth. The latter was verified for a small subset of spots by focusing on the bottom of the pit. Calibration standards (NIST 610 and 612) were ablated in duplicate at the start and end of each slide of 12–15 otoliths with a further replicate midway through each sequence. Based on the method described in Sagar and Palin [16], the reduction of the raw count rate data to element ratios utilised an offline Excel spreadsheet and known values for the glasses [17]. Any drift in the mass bias (typically less than 3%) was corrected prior to statistical analysis.

### Statistical Analyses

Otolith chemical composition was analysed with respect to sex, length, age, sampling time and sampling location. Before analysis all covariates were investigated with respect to colinearity. Statistical analyses were carried out on otolith core and edges separately, the core representing the environment of the first period in the fish life and the edge the environment near the location where it was caught. An 80µm sample from the edge of an otolith from a 4 year austral hake represents approximately 1.5 months of growth as determined from measurements taken from the sections utilised in this study. In total, 30 trace elements were detected however only five (^138^Ba, ^88^Sr, ^118^Sn, ^23^Na, and ^24^Mg) in the otoliths edges and (^138^Ba, ^88^Sr, ^118^Sn, ^24^Mg, and ^55^Mn) in the cores had concentrations significantly above the detection limits. Subsequently all elements used in analyses are presented as ratios to ^44^Ca.

Statistical analyses were performed in R and PERMANOVA+ for PRIMER.

#### Otolith Edge

The impacts of sampling site, sex, length and age on individual trace element ratios in otolith edges were investigated using GLMs. Where necessary data were transformed using Box-Cox transformation to ensure normality.

Partial correlation models were applied to concentration of each element to test for the impact and significance of the individual covariates after partialling out the effect caused by the others.

Canonical analysis of principal coordinates (CAP) using PERMANOVA+ for PRIMER statistical software [18] was used to investigate whether there were significant differences in trace element compositions between sampling sites. CAP is a flexible constrained ordination procedure which allows any distance or dissimilarity measure to be used, while also taking into account correlation structures among variables in the response data. Here CAP was used as a canonical discriminant analysis of principal coordinates using the Bray-Curtis similarity index as the distance measure. Tests of hypothesis for differences between groups and the correct classification were conducted by permutation 10,000 procedures on the canonical test statistic.

#### Otolith Core

The impacts of age on individual trace elements were investigated using GLMs, where necessary element concentrations were transformed to ensure normality.

The extent to which two or more populations of fish exit was investigated using cluster analysis. Ward's cluster algorithm on a distance matrix using the Kulczynski coefficient (Legendre and Legendre, 1998) [19] was initially applied to detect multivarate patterns in the otolith core concentrations. In order to detect the range of otolith concentrations in each cluster identified, a finite mixture analysis was applied for each trace element following Grün [20] using Markov chain Monte Carlo (MCMC) algorithms. The finite mixture model has a hierarchical structure of probability distributions. As the main driver in separation between the resulting clusters was Sr/Ca, Bayesian finite mixture models with 10,000 MCMC iterations were applied to test for two and three separate populations.

### Results

Initial investigation of covariates identified a strong correlation between sampling site and sampling time, therefore the latter was not investigated further.

**Fig 2:**
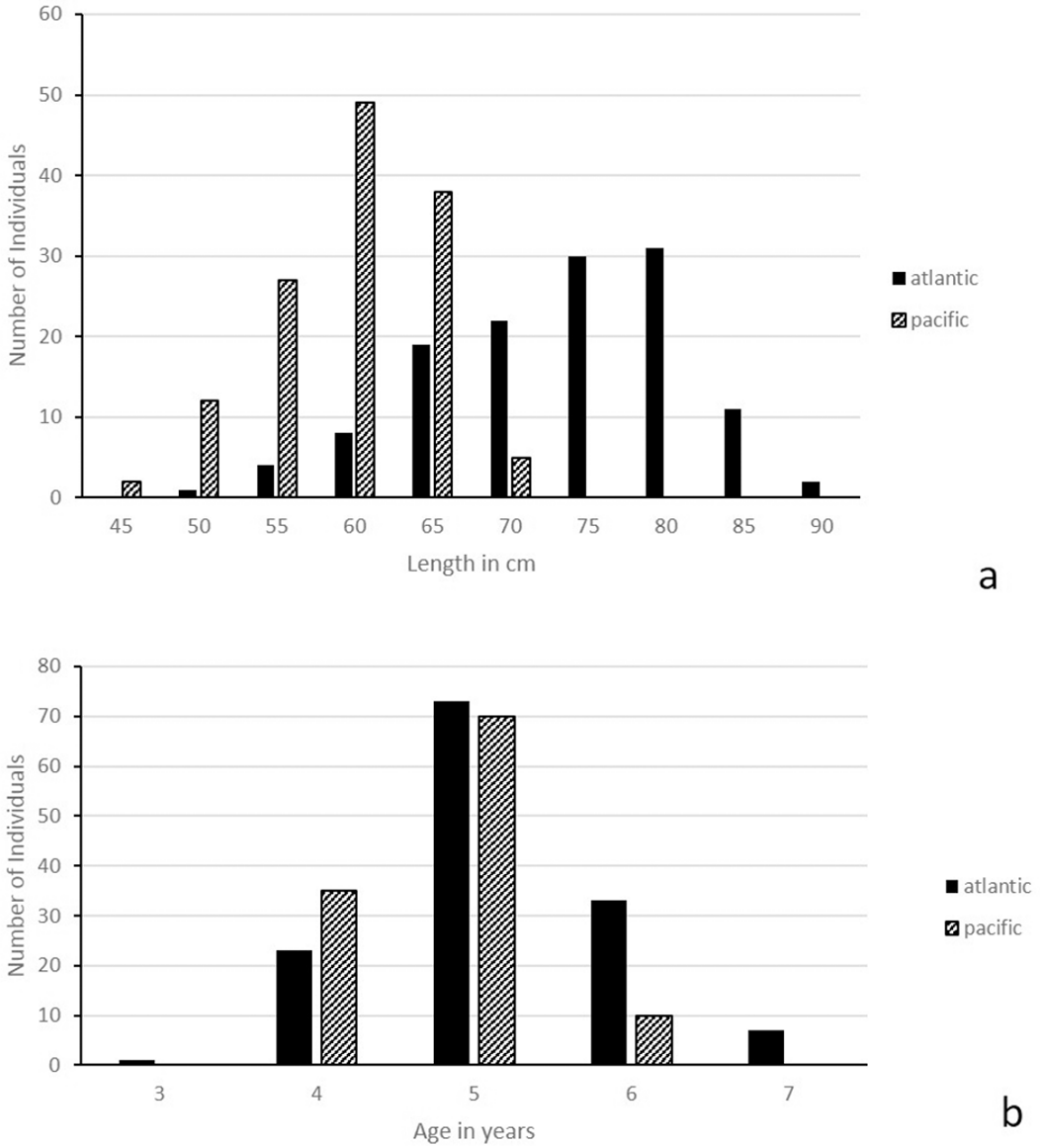
Length frequency (a) and age frequency (b) of Chilean and Falkland Islands samples.

Fish size in samples ranged from 47–88 cm L_T_ and 43–67 cm L_T_ for the Atlantic and Pacific, respectively. Corresponding ages were 4–7 and 3–6 years, respectively (Fig 2). Fish size and sampling site were correlated due to a sampling bias (Table 1, Table 2) with Atlantic samples displaying a larger size range than Chilean samples.

**Table 2:** Results of univariate analysis (GLM) of element concentrations in the edge of otoliths in Chilean and Falkland Islands' samples. Analysis was by Partial Correlation partialling out the effect of other covariates on the impact of a single covariate. Prior to analysis concentrations were transformed by x^λ^ F statistic dependent on 263 DF (Degrees of Freedom).

Element	Variable	F	p
Ba	Sex	13.41	0.0003
	Site	4.498	0.0349
	Length	0.185	0.668
Na	Sex	2.494	0.115
	Site	4.561	0.034
	Length	0.153	0.696
Mg	Sex	0.104	0.747
	Site	1.27	0.261
	Length	0.004	0.951
Sr	Sex	17.67	0.000
	Site	9.62	0.002
	Length	5.75	0.017
Sn	Sex	1.545	0.215
	Site	1.455	0.229
	Length	0.292	0.589

Ratios of Sr/Ca, Na/Ca and Ba/Ca in the otolith edges showed significant variation between sites (Table 2), Ba/Ca and Sr/Ca showed significant variation between sexes (Table 2), while Sr showed also significant impact of size. After partialling out the effect of sex and size ratios of all three elements showed significant impact of sampling location (Table 2). Ratios of Mg and Sn showed no significant impact for either of the covariates tested for (Table 2).

**Fig 3:**
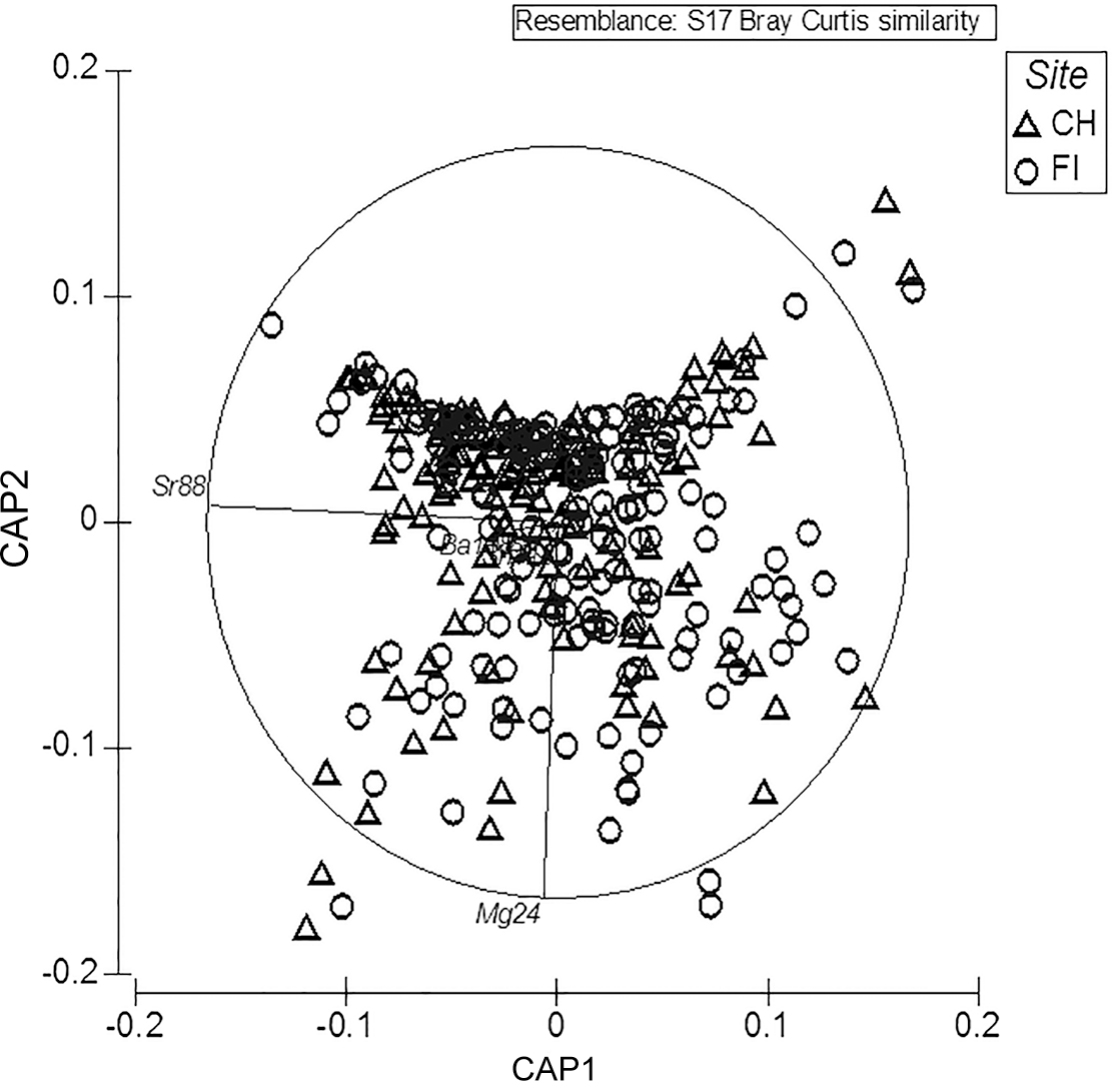
Ordination of Principal Coordinates of otolith edge elements of samples from both sampling sites. FI = Falkland Islands; CH = Chilean samples.

Non-transformed edge ratios of Mg/Ca, Sr/Ca, Ba/Ca, Sn/Ca and Na/Ca were included in the multivariate analysis. CAP showed a significant separation in element ratios between fish from Pacific and Atlantic (δ12 = 0.02141, P = 0.013) using a permutation test with 10,000 permutations. Strongest impacts were seen for Sr/Ca and Mg/Ca (Fig 3). However, the overall correct classification by group was poor with only 57.6% of the otoliths being assigned to the location they were collected from, highlighting poor discrimination.

**Fig 4:**
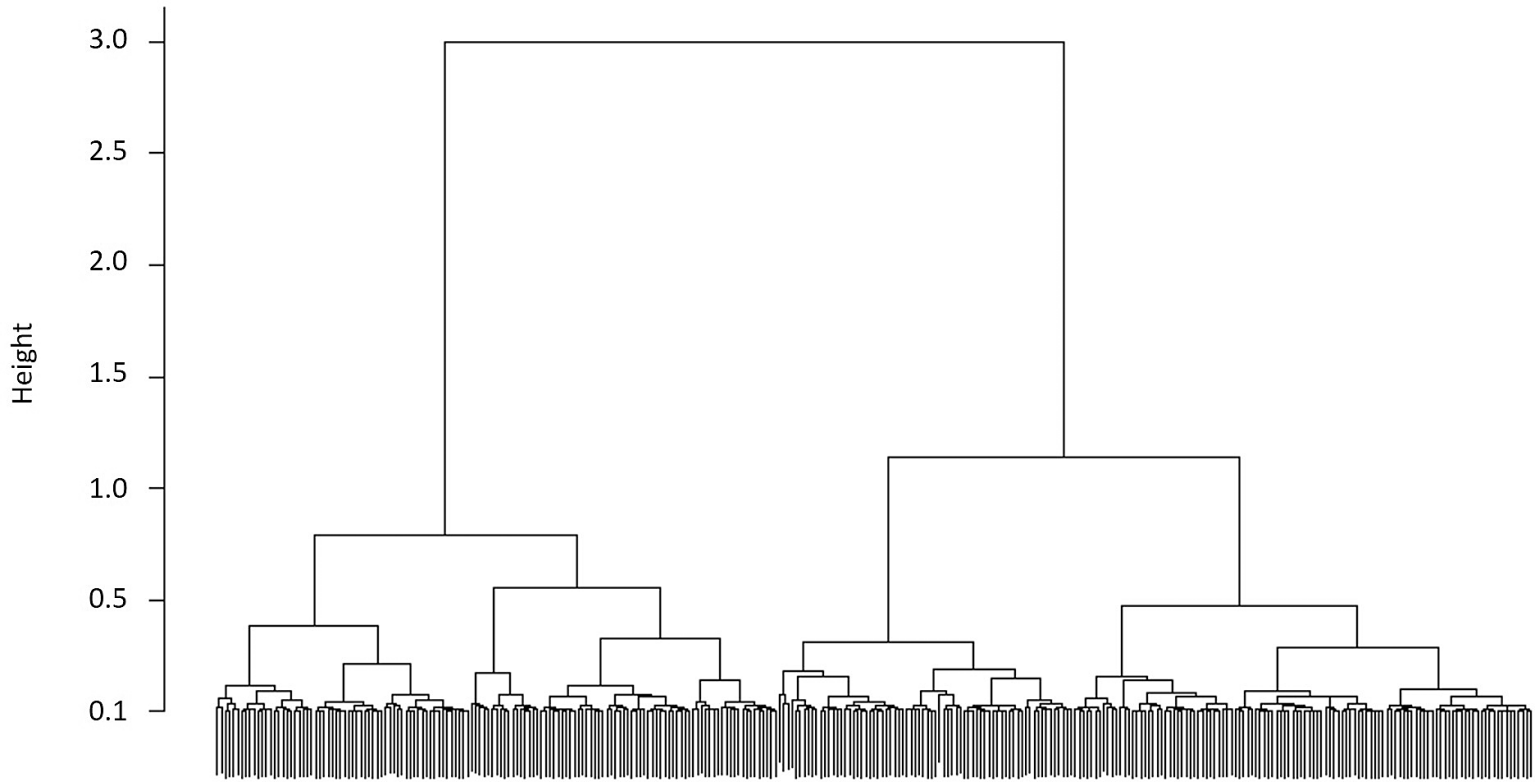
Dendrogram of Ward’s cluster results of otolith core elemental ratios.

**Fig 5:**
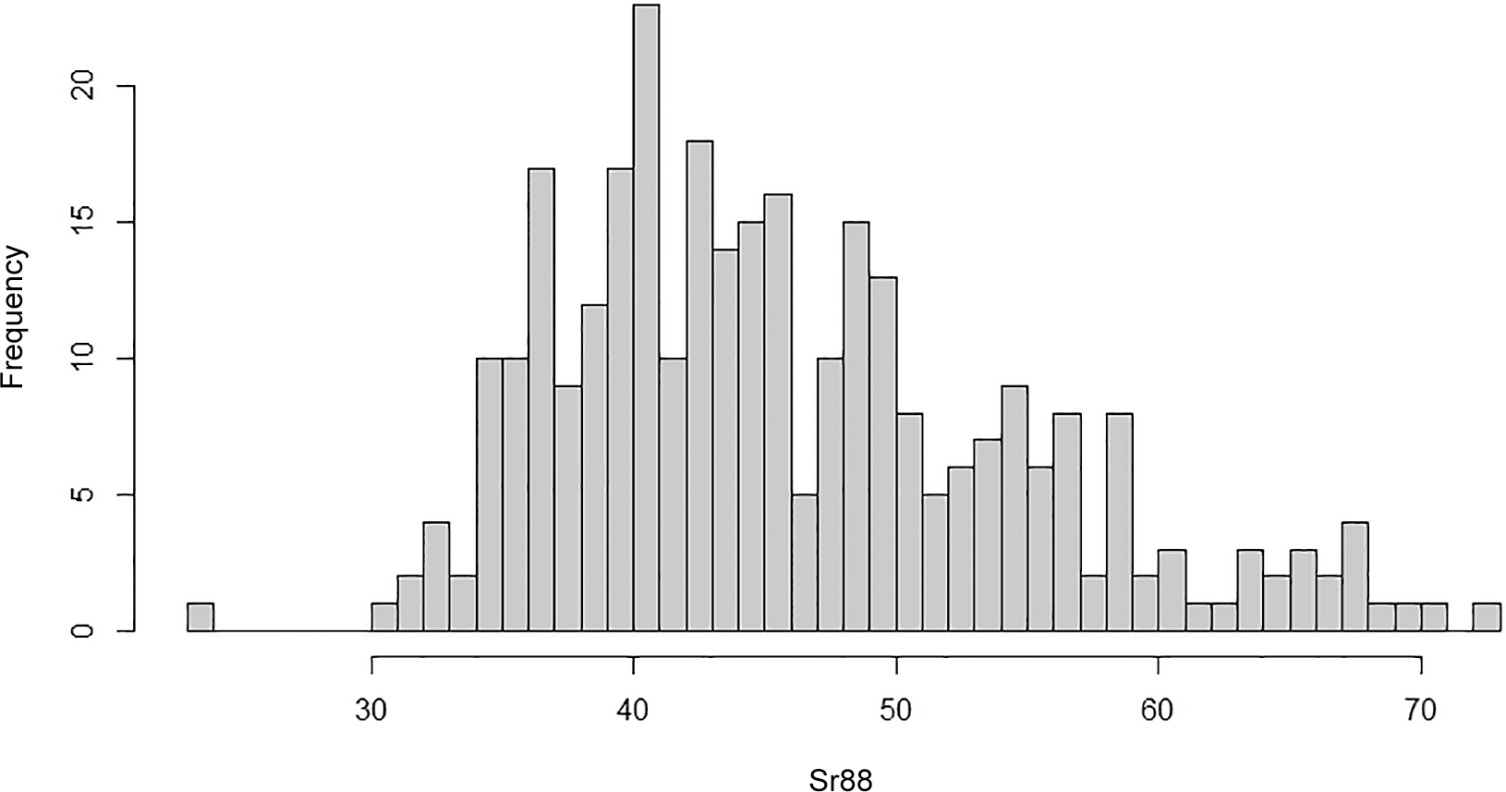
Frequency distribution of Sr/Ca ratios in otolith cores.

Age was non-significant in core ratios of Mg/Ca, Mn/Ca, Sr/Ca and Sn/Ca (Table 3) while it was significant in ratios of Ba (F = 2.012, p = 0.045). Agglomerative clustering analysis, on core concentrations, based on Ward’s similarity/distance metric suggested that it was possible to identify two groups (Table 4, Fig 4). Table 5 illustrates the elemental concentrations by cluster. Perceived clustering of the core concentrations in Ward’s clustering algorithm was largely due to the wide spread of Sr/Ca ratios in the otoliths core (Fig 5).

**Table 3:** Significance of univariate GLM results on 269 DF testing for significant differences in core concentrations between ages. Prior to analysis concentrations were log(x) transformed.

Trace element	Age	
	F-value	p
Ba	2.012	0.045
Mg	-0.402	0.688
Sr	1.515	0.131
Sn	0.663	0.508
Mn	-0.259	0.797

**Table 4:** Cluster membership of core concentrations of individuals by sampling site.

Cluster	Atlantic	Pacific	Total
1	86	94	180
2	54	34	88

**Table 5:** Element concentrations in the core for all individuals combined the individuals in cluster 1 and the individuals in cluster 2.

	Total		Cluster 1		Cluster 2	
	Mean	Std	Mean	Std	Mean	Std
Mg	0.573	0.188	0.565	0.187	0.591	0.188
Mn	0.036	0.024	0.032	0.020	0.046	0.029
Sr	46.348	8.866	50.719	7.399	37.412	2.920
Sn	0.018	0.005	0.018	0.006	0.017	0.004
Ba	0.089	0.08	0.092	0.089	0.081	0.055

**Fig 6:**
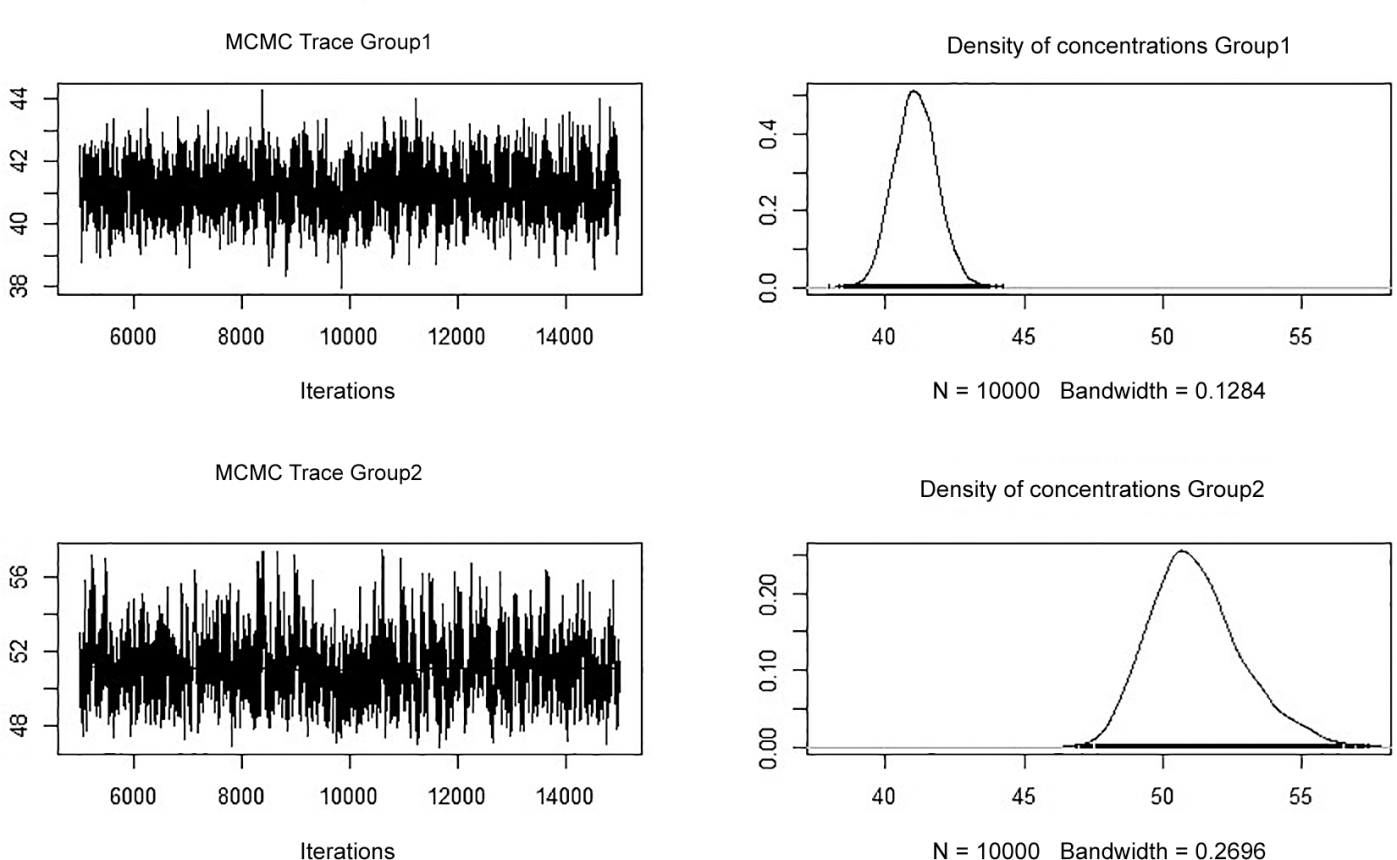
Distribution of µ in the Sr/Ca core trace element composition in the two distributions identified by finite mixtures with 10,000 MCMC runs.

No evidence was found for correlations between the raw element ratios using the Spearman test (Table 6). Gaussian finite mixtures using MCMC method provided evidence that Sr/Ca ratios form two separate distributions with significantly different mean (µ) values (Table 6, Fig 6), while ratios of other elements showed no evidence of the presence of two or more populations.

**Table 6:** Mean values, standard deviation and 95% confidence intervals after 10,000 MCMC iterations for µ of the two identified populations in the ^88^Sr concentrations in otolith cores.

Mean	Standard Deviation	Lower 95% C.I.	Upper 95% C.I.
41.10	0.903	39.64	42.98
51.26	2.114	47.72	55.71

## Discussion

Understanding the spatial structure of fish stocks and the connectivity within and between stocks is increasingly important in underpinning sustainable fisheries management. A number of different methods have been used to investigate spatial structure including mark-recapture experiments, pop-up satellite archival tags (PSAT), electronic data storage tags, parasite infections, otolith shape and genetics. In addition, otolith chemistry has provided great potential for reconstructing movements of fish over their lifetime. The basic assumption is that as the otolith grows, chemical markers from the environment are incorporated into the otolith microstructure resulting in a ‘signature’ that reflects physicochemical properties of the environment in which it was formed [21–24].

Trace elemental signatures in the edge of otoliths accumulate in the final part of the fish’s life before natural death or capture and have been used to trace migrations [25,26]. For austral hake, if trace element signatures in the edge differ between sampling sites this might indicate a close affinity of fish to stay within their own group suggesting a differential migratory behaviour between Atlantic and Pacific fishes. If the variation between groups cannot be satisfactorily discriminated based on the signature in the edges of their otoliths, it might indicate either that the two groups belong to the same stock or, alternatively, that they belong to separate stocks that utilize the same feeding grounds. Another potential driver is that the chemistry of the water masses was not sufficiently different to generate differences in otolith signatures for fish separated by 100s of km. However, the latter is unlikely as the water masses in the Falkland Islands and Chile are of different origin. The Antarctic Circumpolar Current gives rise to the Falklands Current [27] and the West Wind Drift bifurcation (WWD-B) results in the northern flowing Humboldt and south flowing Cape Horn currents [28]. For example Ashford et al [29] found that Patagonian toothfish (*Dissostichus eleginoides*) caught in the Falkland Islands showed heterogeneity in core otolith signatures. Their conclusions were that toothfish available to trawlers in the Falkland Islands likely derived from separate spawning grounds on the Burdwood Bank and from south of the Diego Ramirez Islands in southern Chile. This being a reflection of the different environmental conditions at each area and therefore different elemental signatures in the cores.

In this study discrimination between sites in otolith edges was not possible due to poor classification in CAP (57.6%), although there was a slight detectable variation between samples from both sites. As a strong correlation existed between site and size (Table 3), with Falkland Island specimens reaching larger sizes than their Chilean counterparts (Table 1), the detected variation might not originate from differing site but rather from a larger size. Larger fish have stronger swimming skills and hence can migrate faster and farther than smaller fish. Faster fish arrive at their feeding grounds earlier after spawning and as they are there longer they will accumulate area specific signatures more strongly.

A similar pattern was observed in southern blue whiting and Patagonian toothfish [25,28] where samples collected from the Pacific and Atlantic Oceans could not be distinguished using trace element signatures taken from the otolith edges. This may indicate that austral hake is migratory and moves between the Pacific and Atlantic Oceans. Other explanations could include i) the otolith edge sample incorporates a substantial time period of the fish´s life before capture (i.e. not at the capture location), during which the population was mixing; or prior to dispersal to spatially distant capture locations, ii) the otolith edge samples reflect the capture locations and the water properties are very similar between capture locations resulting in similar edge chemistry. These are unlikely as we are confident that samples taken at the edge of the otolith represent the period prior to capture and the fish were collected from two very different ocean current systems. Exposure to oceanographic properties characteristic of water masses and circulation leave a different signature that fish carry with them [29, 30]. In contrast trace elemental signatures in hoki otolith edges exhibited three signatures (clusters), in all of the sampling sites conducted by Schuchert et al [26]. This indicated that this species is highly migratory and does not show close affinities to their feeding grounds. The Schuchert et al [26] study supports the conclusion of the analyses of genetic data that hoki is a single (intermixed) stock from the southwest Atlantic [31] [32], and added further weight to their possible migrations between the two oceans around Cape Horn and via the Strait of Magellan [33].

Trace element signatures in the otolith core accumulate during the spawning /early larval periods of life and therefore provide insight to environmental variations at spawning /early larval sites [34]. Our data suggest one spawning region in the Pacific which supports the work of Payá and Ehthardt [14] and Bustos et al [15]. A minor southern spawning area at 52° - 54°S may have existed in the 1980’s, but there is no evidence that this area exists today (Páya pers. comm.). Interestingly, there is a large range in Sr/Ca ratios in the cores which may indicate complex salinity structuring in the austral hake spawning and/or larval grounds. Major and minor otolith constituents have been described as valid environmental indicators; this is particularly true of Sr and Ba. Sr in water follows a quasiconservative distribution, allowing otolith Sr/Ca ratios to act as a powerful marker of movement across salinity gradients [23]. Consequently, Sr/Ca ratios are routinely applied to reconstructing migrations of diadromous fish [35–43]. The interpretation of Sr/Ca signals requires caution, however, as considerable interspecific variation in Sr uptake rates exist [44,45]. In addition, the potential exists for vateritic inclusions to be misidentified as freshwater excursions [43] and possibly confounding effects of temperature and physiology [33,46–48].

The spawning and nursery grounds for austral hake, until relatively recently, were not well known. Bustos et al [15] used oceanographic, egg and larval surveys conducted in the Chilean fjords (43°30’S– 47°S) between 1995 and 2002 to describe the utilisation of southern Chile’s Patagonian Inner Sea (PIS) as a spawning site for austral hake. They found large patches of eggs with undeveloped embryos, eggs in late development and large densities of larvae (up to 385m-2) inside the fjord system, in the PIS, during the austral spring. Eggs and larvae <9 mm (preflexion) were rarely detected in open and ocean influenced waters. Bustos et al [15] concluded that the higher frequency of small larvae inside the fjords of the PIS, and the presence of postflexion larvae outside were indicative of the fjords being used as nursery grounds. Additionally, young of the year have only been reported in the PIS [11], particularly in and adjacent to Aysen Fjord and the Gulf of Ancud. In the PIS rainfall (2.5 m^-yr^ Strub et al [49]) and freshwater run off from the Andes generate a low salinity (<10–30) surface layer of approximately 20 m depth resulting in a strong halocline and well defined stratification [50,51,52]). Below the surface layer the water column is near homogenous [15]. This makes for contrasting physical conditions in the PIS and adjacent oceanic waters where this halocline is absent [15].

In addition, a recent study by Medina et al [53] using fatty acid composition of gonad, liver, and muscle tissues from female austral hake found differences between individuals collected from internal (PIS) and offshore waters (oceanic), suggesting that their main food sources and overall trophic webs were different. They suggested that that these groups of fish reside separately in each zone during the winter season. Niklitschek et al [54] also highlighted the potential importance of the PIS, one of the largest and most complex estuarine systems in the world, for *M*. *magellanicus* and other ground fish. Their results support that *M*. *magellanicus* does not depend on the estuarine systems in the region to complete its life cycle, but use them as a “portfolio” of nursery and feeding grounds. It is likely that the high variation in Sr/Ca ratios in the otolith cores of austral hake is indicative of this complex salinity structuring in the spawning and larval grounds.

There must be some caution applied here as there are potentially confounding effects of temperature and physiology on the uptake of Sr in fish otoliths. Panfili et al [55] found, based on experimentation using tilapia (*Sarotheron melanotheron heudelotii*), that in most fish tested (*c* 80%) the relationship between Sr/Ca and salinity was positive but non-linear. But 20% of the individuals from control and experimental treatments showed consistently low Sr/Ca ratios, irrespective of salinity. They concluded that there was high variability between individuals in the regulation of Sr incorporation. Further work is required to confirm this with the collection of otoliths from early stages inhabiting the fjord system in order to examine the Sr/Ca signatures coupled with an investigation into δ^18^O/δ^16^O ratios in the cores to confirm any temperature variations.

Data collected and analysed in our study and by other researchers [7,14,15] allow for a conceptualised model of austral hake life history. It assumes a single population that spawns in austral winter/spring in coastal ocean waters of Chile, with a proportion of the eggs and larvae transported to the inner waters of the fjord system (PIS). Juveniles and young adults disperse to adjacent coastal ocean waters to feed. At some point they return to spawn. An unknown proportion of adult fish, after spawning, then migrate south via Cape Horn to Atlantic waters to feed. This could have serious implications to regional fisheries management and further work is required to elucidate the proportion of the population utilising Argentine and Falkland waters as feeding grounds as part of their life cycle. Further investigations should also ascertain whether Atlantic fish make annual spawning migrations or whether there are specific age, or more likely size, factors to these migrations. The latter could be determined by running sample transects from the otolith core to the edge. This would provide much more information to examine whether fish moved among water masses during their lifetime.
